# Acid-Sensing Ion Channels’ Immunoreactivity in Nerve Profiles and Glomus Cells of the Human Carotid Body

**DOI:** 10.3390/ijms242417161

**Published:** 2023-12-05

**Authors:** Graciela Martínez-Barbero, Yolanda García-Mesa, Ramón Cobo, Patricia Cuendias, Benjamín Martín-Biedma, Olivia García-Suárez, Jorge Feito, Teresa Cobo, José A. Vega

**Affiliations:** 1 Grupo SINPOS, Departamento de Morfología y Biología Celular, Universidad de Oviedo, 33006 Oviedo, Spain; gracielamartinezbarbero@gmail.com (G.M.-B.); garciamyolanda@uniovi.es (Y.G.-M.); ramoncobodiaz@gmail.com (R.C.); cuendiaspatrica@uniovi.es (P.C.); garciaolivia@uniovi.es (O.G.-S.); 2 Servicio de Otorrinolaringología, Hospital Universitario Marqués de Valdecilla, 39008 Santander, Spain; 3 Departamento de Cirugía y Especialidades Médico-Quirúrgicas, Universidad de Santiago de Compostela, 15782 Santiago de Compostela, Spain; benjamin.martin@usc.es; 4 Servicio de Anatomía Patológica, Complejo Asistencial Universitario, 37007 Salamanca, Spain; jfeito@saludcastillayleon.es; 5 Departamento de Cirugía y Especialidades Médico-Quirúrgicas, Universidad de Oviedo, 33006 Oviedo, Spain; teresacobo@uniovi.es; 6 Instituto Asturiano de Odontología, 33006 Oviedo, Spain; 7 Facultad de Ciencias de la Salud, Universidad Autónoma de Chile, Providencia 7500912, Región Metropolitana, Chile

**Keywords:** carotid body, glomus cells, nerves, acid-sensing ion channels, immunohistochemistry, human

## Abstract

The carotid body is a major peripheral chemoreceptor that senses changes in arterial blood oxygen, carbon dioxide, and pH, which is important for the regulation of breathing and cardiovascular function. The mechanisms by which the carotid body senses O_2_ and CO_2_ are well known; conversely, the mechanisms by which it senses pH variations are almost unknown. Here, we used immunohistochemistry to investigate how the human carotid body contributes to the detection of acidosis, analyzing whether it expresses acid-sensing ion channels (ASICs) and determining whether these channels are in the chemosensory glomic cells or in the afferent nerves. In ASIC1, ASIC2, and ASIC3, and to a much lesser extent ASIC4, immunoreactivity was detected in subpopulations of type I glomus cells, as well as in the nerves of the carotid body. In addition, immunoreactivity was found for all ASIC subunits in the neurons of the petrosal and superior cervical sympathetic ganglia, where afferent and efferent neurons are located, respectively, innervating the carotid body. This study reports for the first time the occurrence of ASIC proteins in the human carotid body, demonstrating that they are present in glomus chemosensory cells (ASIC1 < ASIC2 > ASIC3 > ASIC4) and nerves, presumably in both the afferent and efferent neurons supplying the organ. These results suggest that the detection of acidosis by the carotid body can be mediated via the ASIC ion channels present in the type I glomus cells or directly via sensory nerve fibers.

## 1. Introduction

The peripheral chemoreceptors, especially the carotid body (CB), are major sensors known to detect changes in arterial blood oxygen (hypoxemia), carbon dioxide (hypercapnia), and pH (acidosis). The mechanisms by which the CB senses the first two are well known, while those of acidosis are unclear [1,2,3].

Recent studies revealed that some membrane proteins, including acid-sensing ion channels (ASICs), allow sensory neurons to monitor tissue acidosis [4]. ASICs form a family of amiloride-sensitive, proton-gated channels belonging to the superfamily of degenerin/epithelial sodium channels (DEG/ENa^+^C), which are phylogenetically preserved from invertebrates to mammals [5] and are widely expressed, especially in the nervous system [6]. At present, six ASIC isoforms (ASIC1a, 1b, 2a, 2b, 3, and 4) encoded by four genes (*Accn1*, *Accn2*, *Accn3*, and *Accn4*) have been identified in vertebrates [7,8,9]. ASICs participate in several physiological roles, including mechanosensation [10], nociception [11], and chemosensation [9,12]. Here we focused on chemosensation, although the force and necessary pH must act together to induce ASIC activity [13]. 

ASICs can be activated by a drop in extracellular pH below 7.0 and triggered by non-proton ligands at physiological pH levels [14]. They are sensitive to changes in pH because proton ligands bind to their extracellular domains, inducing channel activation, which generates currents via sodium transit across membranes in a voltage-insensitive manner [7,15]). The pH sensitivity of ASICs decreases in the following order: ASIC3  ≥  ASIC1a  >  ASIC1b > ASIC2a [16].

Regarding the CB, it has been proposed that extracellular acidosis and a drop in intracellular pH activate chemoreceptors through ASIC channels [17]. Proton-gated currents with biophysical and pharmacological properties comparable to those of ASICs, as well as expression of ASIC1 and ASIC3, have been reported in rat glomus type I cells mediating the response to pH and hypoxia [18,19]. Nevertheless, the roles of ASICs in the CB are unclear, since deletion of *Asic3* in mice disrupts the increase in intracellular Ca^2+^ evoked in response to extracellular acidification but not to hypoxia [19]; in ASIC1-, ASIC2-, and ASIC3-knockout mice, this does not significantly alter hypercapnic or hypoxic ventilatory responses [20]. Independently of the chemosensory cells of the CB, the afferent nerve terminals emanating from the petrosal ganglia neurons could also directly sense variations in pH, as they have been shown to express some ASIC isoforms [17,21]. 

As far as we know, no data exist on the distribution of ASIC channels in the human CB, either in the glomic cells or in the afferent nerve terminals. Therefore, the present research was designed to analyze the occurrence of ASIC subunits using immunohistochemistry and to determine whether they are expressed in nerves, glomus cells, or both. The study aims to serve as a baseline in future studies of pathologies in which the CB is involved, such as arterial hypertension [3,22,23] or sleep apnea syndrome [24,25].

## 2. Results

The glomus lobes arre arranged in the vicinity of, and around, the blood vessels and are separated from each other by connective tissue septa of different thicknesses. Each lobe, in turn, consists of a variable number of glomeruli made up of type I and type II cells. The identification of the two main types of glomic cells was established in serial sections for the immunohistochemical detection of NSE and SYN, which mark type I, and of S100P, which selectively marks type II cells [26,27] (Supplementary Material Figures S1 and S2). Antibodies against NFP (phosphorylated 200 kDa subunit) and NSE were used to demonstrate the innervation of the carotid body. The anti-NFP antibody used strongly immunostained peripheral nerve fibers but has the disadvantage that it does not allow for differentiation between afferent and efferent nerve fibers [28]. But as already mentioned, the profile of the innervation of the CB is completed with the results on anti-SYN and anti-NSE antibodies (Supplementary Material Figures S3 and S4). 

**Figure 1:**
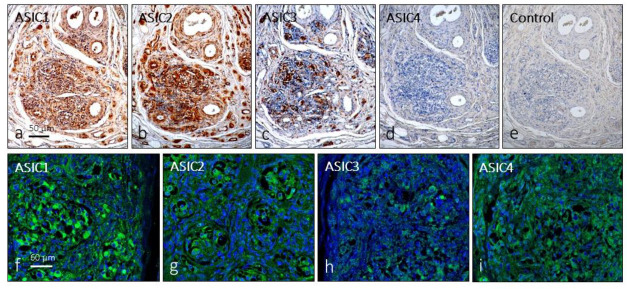
Expression of immunoreactivity in serial sections (**a**–**d**) and immunofluorescence (**f**–**i**) for ASIC ion channels in the human carotid body. (**e**): negative control omitting the primary antibody in incubation. The images in (**f**–**i**) were obtained using a 60/1.25 oil lens with a 1.XY pinhole resolution of 156 nm and a Z resolution of 334 nm. Scale bar in a applies for (**a**–**e**); scale bar in (**f**) applies for (**f**–**i**).

The first approach, in serial sections, showed that distinct cell populations displayed immunoreactivity for all ASIC subunits, although for ASIC4, immunostaining was very weak or not detectable in some cases; negative controls omitting the primary antibody at incubation resulted in the total absence of immunoreactivity (Figure 1a–e). Furthermore, ASIC1 and ASIC2 were detected in blood vessels and in some nerve profiles; however, in the glomus glomeruli, it is not possible to establish precisely which cells express these proteins. The results of immunofluorescence confirmed the results (Figure 1f–i). 

**Figure 2:**
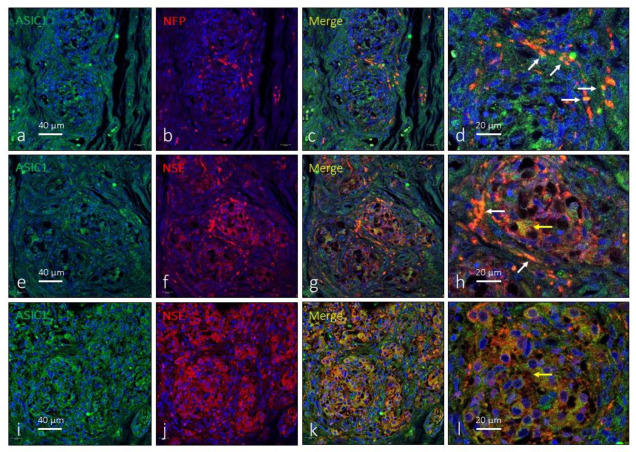
Immunofluorescence detection of the ASIC1 ion channel in the human carotid body. ASIC1 immunoreactivity was observed in clusters of type I glomus cells scattered throughout the organ (yellow arrows) and nerve profiles (white arrows). Serie (**a**–**d**) shows the localization of ASIC1 in NFP positive profiles. Series (**e**–**h**) and (**i**–**l**) show the localization of ASIC1 in type I glomus cells and nerves and correspond to two different subjects (aged 38 and 68 years, respectively). NFP: neurofilament protein, NSE: neuron-specific enolase. Lens: 60/1.25 oil; 1.XY pinhole resolution: 156 nm; Z resolution: 334 nm. Scale bar in (**a**) applies for (**a**–**c**); scale bar in (**e**) applied for (**e**–**g**); scale bar in (**i**) applied for (**i**–**k**).

The antibody against ASIC1 used here recognized the two isoforms of this protein (suppliers notice). ASIC1 immunoreactivity was detected broadly in the CB, apparently in subpopulations of type I glomus cells (Figure 2e–l) and nerve terminals (Figure 2a–d). However, the immunoreaction was not the same in all glomeruli nor was it present in all cells. ASIC1-positive nerve profiles were observed both in the peripheral part of the organ as well as in nerves localized in the connective septa between lobules and/or glomeruli. Synaptic contacts between nerves and type I glomus cells were not observed using light microscopy. The area occupied by the ASIC1-specific immunoreaction was 43.2 ± 3.6% of the total section, and the area occupied by the emergence of both glomic cells and nerves was 2.6 ± 0.3% of the total section (Table 1). 

**Figure 3:**
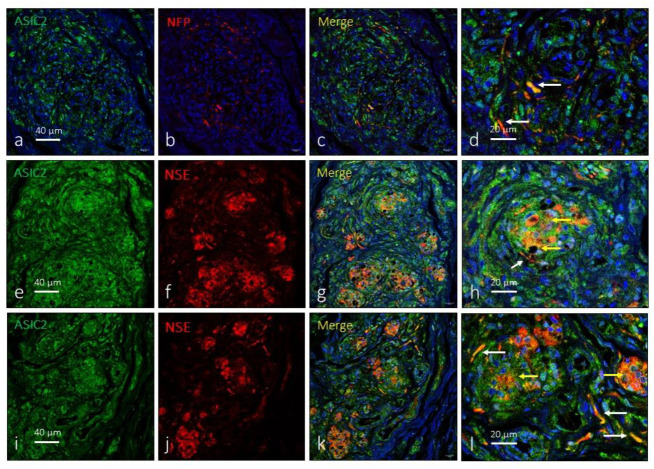
Immunofluorescence detection of the ASIC2 ion channel in the human carotid body. ASIC2 immunoreactivity was observed in clusters of type I glomus cells scattered throughout the organ (yellow arrows) and nerve profiles (white arrows). Serie (**a**–**d**) shows the localization of ASIC2 in NFP positive profiles. Series (**e**–**h**) and (**i**–**l**) show the localization of ASIC2 in type I glomus cells and nerves and correspond to two different subjects (aged 41 and 68 years, respectively). NFP: neurofilament protein, NSE: neuron-specific enolase. Lens: 60/1.25 oil; 1.XY pinhole resolution: 156 nm; Z resolution: 334 nm. Scale bar in (**a**) applies for (**a**–**c**); scale bar in (**e**) applied for (**e**–**g**); scale bar in (**i**) applied for (**i**–**k**).

As for ASIC1, the anti-ASIC2 antibody used recognized the two subunits of the protein. ASIC2 was also widely expressed in the carotid body. A large proportion of type I glomus cells showed ASIC2-immunoreactivity (Figure 3e–l), and positivity was also detected in nerve terminals (Figure 3a–d) located, preferably, on the surface of the organ and not between the glomeruli. The area occupied by the ASIC2-specific immunoreaction was 61.1 ± 2.8% of the total section, and the area occupied by the emergence of both glomic cells and nerves was 1.9 ± 0.2% of the total section (Table 1). 

**Figure 4:**
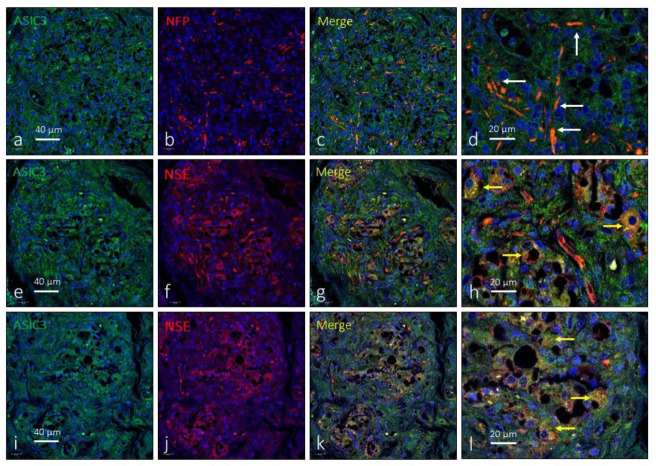
Immunofluorescence detection of the ASIC3 ion channel in the human carotid body. ASIC3 immunoreactivity was observed in clusters of type I glomus cells scattered throughout the organ (yellow arrows) and nerve profiles (white arrows). Serie (**a**–**d**) shows the localization of ASIC3 in NFP positive profiles. Series (**e**–**h**) and (**i**–**l**) show the localization of ASIC3 in type I glomus cells and nerves and correspond to two different subjects (aged 38 and 52 years, respectively). NFP: neurofilament protein, NSE: neuron-specific enolase. Lens: 60/1.25 oil; 1.XY pinhole resolution: 156 nm; Z resolution: 334 nm. Scale bar in (**a**) applies for (**a**–**c**); scale bar in (**e**) applied for (**e**–**g**); scale bar in (**i**) applied for (**i**–**k**).

The expression of ASIC3 in the human CB was low and was only detected in small clusters of type I glomus cells (Figure 4e–l). Occasionally, images suggestive of the presence of ASIC3 were observed in the interlobular nerve profiles (Figure 4a–d). The area occupied by the ASIC3-specific immunoreaction was 9.7 ± 2.4% of the total section, and the area occupied by the emergence of both glomic cells and nerves was almost identical at 2.3 ± 0.3% of the total section (Table 1). 

**Figure 5:**
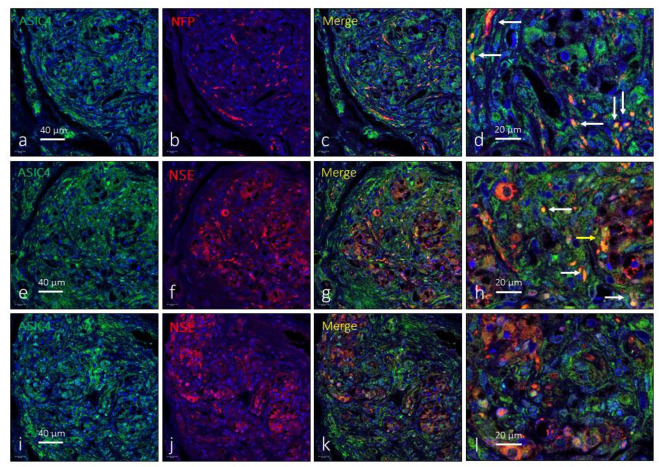
Immunofluorescence detection of the ASIC4 ion channel in the human carotid body. ASIC4 immunoreactivity was observed in clusters of type I glomus cells scattered throughout the organ (yellow arrows) and nerve profiles (white arrows). Serie (**a**–**d**) shows the localization of ASIC4 in NFP positive profiles. Series (**e**–**h**) and (**i**–**l**) show the localization of ASIC4 in type I glomus cells and nerves and correspond to two different subjects (aged 46 and 52 years, respectively). NFP: neurofilament protein, NSE: neuron-specific enolase. Lens: 60/1.25 oil; 1.XY pinhole resolution: 156 nm; Z resolution: 334 nm. Scale bar in (**a**) applies for (**a**–**c**); scale bar in (**e**) applied for (**e**–**g**); scale bar in (**i**) applied for (**i**–**k**).

Finally, the immunoexpression of ASIC4 in the human CB was limited to some isolated type I glomus cells in isolated glomeruli (Figure 5e–l) as well as to nerve profiles (Figure 5a–d). The area occupied by the ASIC4-specific immunoreaction was 2.4 ± 2.1% of the total section, and the area occupied by the emergence of both glomic cells and nerves was 2.3 ± 0.9% of the total section. 

The presence of ASIC proteins in type II cells could not be ruled out, since the immunoreaction area of the ASICs was apparently larger than that of type I cells. However, it was not taken into consideration, because of the difficulty of isolating type II cells from connective tissue and because it could also be a case of non-specific background. 

**Figure 6:**
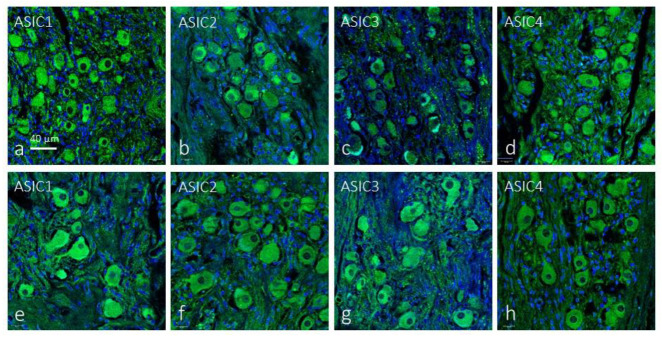
Immunofluorescence detection of the ASIC ion channel in the human carotid petrosal ganglion and superior cervical sympathetic ganglion. Almost all neurons in both afferent and efferent ganglia displayed immunoreactivity for all ASIC proteins. Lens: 60/1.25 oil; 1.XY pinhole resolution: 156 nm; Z resolution: 334 nm. Scale bar applies for (**a**–**h**).

In the sections of both the petrosal ganglia and the superior cervical sympathetic ganglia analyzed, immunoreactivity was found for all ASIC isoforms. The immunoreaction was restricted to the neuronal bodies while the satellite cells were unreactive. In the petrosal ganglia the percentage and size of the immunoreactive neurons were variable for each ASIC protein, whereas apparently all neurons were immunoreactive for each ASIC protein in the sympathetic ganglia (Figure 6 and Table 2). 

## 3. Discussion

It is now accepted that the CB is a multimodal sensor that detects variations in oxygen, carbon dioxide, and pH, as well as low glucose levels, and additionally participates in the regulation of metabolism [29,30]. The detection of blood pH and systemic acidosis is important for the regulation of respiratory feedback and cardiovascular function. 

The present research study was designed to contribute to the knowledge of how the human CB contributes to the detection of variations in pH, to analyze whether it expresses ASIC ion channels, and to know whether these channels are in the cells of the CB itself or in the afferent nerves. We used samples of the human CB from subjects free of disease. To our knowledge, there are no studies on the distribution of ASIC subunits in the human carotid body. Here, we used immunohistochemistry and double immunofluorescence combined with laser confocal microscopy, to accurately identify glomus cells and/or nerves expressing ASICs.

Structurally, the CB consists of two main cell types: chemoreceptor cells and type I cells, organized into tight clusters surrounded by supportive glial cells or type II cells. Type I cells are innervated by afferent fibers, whose soma are located at the petrosal ganglion of the glossopharyngeal nerve, and by efferent fibers emanating from the superior sympathetic cervical ganglion [31,32]. The activation of chemoreceptors in type I cells leads to depolarization and an increase in intracellular Ca^2+^ concentration, followed by the release of multiple neurotransmitters and the synaptic activation of afferent nerve endings [19,33,34,35]. Although type I glomus cells are considered to be chemosensory, the real chemoreceptors are subtypes of sensory neurons in the petrosal ganglion responsible for detecting tissue acidosis. In this context, type I glomus cells can be regarded as accessory cells acting as intermediaries between the blood/extracellular medium/intracellular medium and chemoreceptor neurons [36].

Evidence accumulated over the past 20 years has demonstrated that some membrane proteins allow sensory neurons and sensory cells such as type I glomus cells to monitor tissue acidosis; among those proteins are the different isoforms of ASIC channels. Iturriaga et al. [3], in a general hypothesis about the possible localization of ASIC channels in the carotid body, assume that these may be present in both type I glomus cells and afferent nerve terminals. Our findings are in total agreement with the opinion of these authors, since we have detected these channels in both locations. We observed that mainly ASIC1 and ASIC2, and to a lesser extent ASIC3, and at almost residual levels ASIC4, are expressed in the type I glomus cells of the human carotid body. The expression of ASIC1, ASIC2, and ASIC3 in the rat CB was demonstrated earlier by Tan et al. [28], who found that all these ASIC proteins are expressed in groups of type I glomus cells, and some are co-expressed in the same glomic cells. We did not confirm these results in the human CB, because no co-localization studies were performed; however, when the expression of these proteins was observed in serial sections, it seemed that it was highly possible. 

Regarding the occurrence of ASICs immunoreactivity in nerves, we have regularly observed their presence in the nerves of the carotid body, with very few differences in the innervation density of the positive nerves for each of the four subunits. However, we cannot identify whether they are afferent and/or efferent, since the presence of all ASIC proteins has been detected in the neurons of the petrosal ganglion and the upper cervical sympathetic ganglion. The presence of ASIC isoforms in human spinal and cranial sensory ganglia alike has been little studied. Only Fukuda et al. [21] reported the presence of ASIC channels in the rat petrosal ganglion, specifically ASIC3. Cabo et al. [37] demonstrated the presence of ASIC2 in mechanosensory neurons, as well as small subpopulations of proprioceptive and nociceptive neurons in human lumbar dorsal root ganglia. Nevertheless, the presence of ASIC proteins in the human petrosal ganglion neurons is reported here for the first time. In addition, there is ample functional, physiological, and pharmacological evidence for the presence of all ASIC subunits in rat and mouse spinal ganglia [38,39,40,41]. Thus, the present results suggest that the sensory neurons of the human petrosal ganglion can detect wide ranges of drops in pH, and therefore acidosis, since it expresses all ASIC subunits. Obviously, based on the techniques used, it cannot be said whether these neurons capture variations in intracellular or extracellular pH. Further studies are necessary to try to clarify this issue. Regarding the sympathetic ganglia, there is no reference in the scientific literature that addresses this topic. Only the study by Liu et al. [17] made specific reference to the upper cervical sympathetic ganglion of the rat and affirmed that, under the conditions of their experiment, the ASICs are not modified in the neurons of this ganglion, indirectly suggesting that they do detect some isoforms. However, our work is the first to demonstrate the presence of all ASIC isoforms in sympathetic efferent neurons. The functional significance of these findings, if any, remains to be elucidated. 

In summary, our results demonstrate for the first time the occurrence of ASIC1, ASIC2, ASIC3, and ASIC4 immunoreactivity in the human carotid body, both in the type I glomus cells and in the nerves supplying the carotid body. Therefore, ASICs can generate acid-evoked responses throughout chemosensory cells and afferent nerves. 

## 4. Materials and Methods

### 4.1. Materials

The region of the common artery bifurcation and surrounding tissues were removed from 8 subjects (5 males and 3 females; age range: 38 to 68 years), during the removal of organs for transplantation from subjects who had died in traffic accidents (Hospital Universitario Central de Asturias, Oviedo, Spain). Furthermore, the petrosal ganglion (n = 6) and the sympathetic superior ganglion (n = 4) from the same subjects were dissected and included in the study. The material was obtained in compliance with Spanish Law and the guidelines of the Helsinki Declaration II. 

The tissue samples were cleaned in a 4 °C saline solution, fixed in 10% formaldehyde in 0.1 M PBS, pH 7.4 for 24 h at 4 °C, dehydrated, and routinely embedded in paraffin. For the detection of ASIC1 and ASIC3, deparaffined and rehydrated sections were heated in an Envision FLEX target retrieval solution at a high pH (Dako) at 65 °C for 20 min and then for 20 min at room temperature in the same solution. For the detection of ASIC2, this step was omitted.

### 4.2. Immunohistochemistry

To detect the expression of ASIC proteins in the CB, indirect peroxidase-anti-peroxidase immunohistochemistry was used. Briefly, 10 μm thick sections of paraffin-embedded tissues were mounted on gelatin-coated microscope slides and processed for light microscopy. Rehydrated sections were rinsed in 0.05 M Tris-HCl buffer (pH 7.5) containing 0.1% bovine serum albumin and 0.1% Triton X-100. Endogenous peroxidase activity (3% H_2_O_2_) and non-specific binding (10% fetal calf serum) were blocked, and the sections were incubated overnight in a humid chamber at 4 °C with primary antibodies (Table 3). The antibodies were diluted in a solution of Tris-HCl buffer (0.05 M, pH 7.5) containing 0.1% bovine serum albumin, 0.2% fetal calf serum, and 0.1% Triton X-100. After incubation with the primary antibodies, the sections were rinsed in the same buffer and incubated with Dako EnVision System labeled polymer-HR anti-rabbit IgG or anti-mouse IgG (DakoCytomation, Denmark) for 30 min at room temperature. 

Finally, sections were washed, and the immunoreaction visualized using 3-3′-diaminobenzidine as a chromogen. For control purposes, representative sections were processed as above using non-immune rabbit or mouse sera instead of the primary antibodies or omitting the primary antibodies in the incubation. To visualize structural details, sections were slightly counterstained with hematoxylin and eosin. 

### 4.3. Double Immunofluorescence

Sections were processed for simultaneous detection of ASICs with specific axonal (neurofilament protein—NFP—or neuron-specific enolase—NSE) markers, supportive type II glial cell and Schwann cell (S100 protein—S100P) markers, or type I glomus cells (synaptophysin—Syn) [32,35]. Non-specific binding was reduced via incubation with a solution of 25% calf bovine serum in tris buffer solution (TBS) for 30 min. Sections were incubated with a 1:1 *v*/*v* mixture of polyclonal antibodies against ASIC1, ASIC2, ASIC3, and ASIC4 and monoclonal antibodies against NFP, NSE, S100P, or Syn in a humid chamber overnight at 4 °C. After rinsing with TBS, sections were incubated for one hour with CFL488-conjugated bovine anti-rabbit IgG (diluted 1:200 in TBS; sc-362260, Santa Cruz Biotechnology, Heidelberg, Germany) and then rinsed and incubated again for another hour with CyTM3-conjugated donkey anti-mouse antibody (diluted 1:100 in TBS; Jackson-ImmunoResearch, Baltimore, MD, USA). Both steps were performed in a dark, humid chamber at room temperature. Sections were finally washed, and cell nuclei were stained with DAPI (10 ng/mL). Triple fluorescence was detected using a Leica DMR-XA automatic fluorescence microscope (Microscopía fotónica y Proceso de imágen, Servicios científico-técnicos, Universidad de Oviedo) coupled with Leica Confocal Software (version 2.5; Leica Microsystems, Heidelberg GmbH, Germany), and captured images were processed using the software Image J (version 1.43 g; Master Biophotonics Facility, Mac Master University Ontario; www.macbiophotonics.ca, accessed on 11 January 2021).

For control purposes, representative sections were processed in the same way as described but either not using immune rabbit or mouse sera instead of primary antibodies or omitting primary antibodies during incubation. Furthermore, when available, additional controls were conducted using specifically preabsorbed antisera. Under these conditions, no positive immunostaining was found. 

### 4.4. Quantitative Analysis

A quantitative image analysis was carried out in the carotid body; petrosal ganglia and upper cervical sympathetic ganglia reacted for all ASIC isoforms; detection using an automatic image analysis system (Quantimet 550, Leica, QWIN Program, version 3.5). The percentage of the immunoreactive area for SYN (which identifies type I cells; regarded as 100%) and the merge of SYN + each individual ASIC was determined in 5 sections, for sample, 100 µm apart, measuring 10 randomly selected fields per section (5 mm^2^). Results are expressed as values of mean ± standard error. Furthermore, in the same fields and in 5 additional ones, the density of nerve profiles displaying the merge of NFP + each individual ASIC was evaluated. Results are expressed as the number of nerve profiles/mm^2^. 

On the other hand, the percentage and size (mean diameter in μm) of the immunoreactive neurons for each ASIC isoform were evaluated. Measurements were made on three sections per specimen, 200 μm apart to avoid measuring the same neuron twice, evaluating five randomly selected fields per section (2.5 mm^2^). For the evaluation of the cell body size, only the neuronal profiles with apparent nuclei were considered. The results are expressed as the mean ± standard deviation and refer to the variation among specimens. Due to the low number of sampled corpuscles, no statistical analysis was carried out.

## Figures and Tables

**Table 1:** Percent of the area occupied by ASIC proteins in glomus I cells (merge of ASICs and NSE) and nerves (merge of ASICs and NFP).

	Glomus I Cell	Nerves
ASIC1	43.2 ± 3.6	2.6 ± 0.3
ASIC2	61.1 ± 2.8	1.9 ± 0.2
ASIC3	9.7 ± 2.4	2.3 ± 0.3
ASIC4	2.4 ± 2.1	2.3 ± 0.9

**Table 2:** Percent of neurons positive for ASIC proteins in the petrosal ganglion and superior cervical sympathetic ganglion.

	Petrosal Ganglion	Cervical Superior Sympathetic Ganglion
ASIC1	96.1 ± 9.2	100
ASIC2	98.6 ± 2.3	96.5 ± 1.1
ASIC3	96.4 ± 7.1	100
ASIC4	94.9 ± 5.2	98.1 ± 0.8

**Table 3:** Primary antibodies used in the study.

Antigen	Origin	Dilution	Supplier
ASIC1	Rabbit	1:200	Abcam ^1^
ASIC2	Rabbit	1:200	LifeSpan ^2^
ASIC3	Rabbit	1:100	Abcam ^1^
ASIC4	Rabbit	1:100	LifeSpan ^2^
NSE (clone BBS/NC/VI-H14)	Mouse	1:500	Dako ^3^
NFP (clone 2F11)	Mouse	1:200	Roche ^4^
Synaptophysin (clone 27G12)	Mouse	Prediluted	Leica Biosystems ^5^
S100P	Mouse	1:500	Dako ^3^

^1^ Cambridge, UK; ^2^ Seattle, WA, USA; ^3^ Glostrup, Denmark; ^4^ Vienna, Austria; ^5^ Madrid, Spain. ASIC1: raised against a synthetic peptide to an extracellular domain epitope of human ASIC1, conjugated to immunogenic carrier protein; recognizes both subtype1 and subtype2; ASIC2: raised against a synthetic peptide, specific human; ASIC3: raised against a synthetic peptide to an internal sequence of human ASIC3, conjugated to immunogenic carrier protein; ASIC4: raised against a synthetic peptide from the cytoplasmic domain of human ASIC4, conjugated to immunogenic carrier protein used as an immunogen.

## Data Availability

The data that support the findings of this study are available from the corresponding author upon reasonable request.

## Supplementary Materials

The following supporting information can be downloaded at: https://www.mdpi.com/article/10.3390/ijms242417161/s1.
