# Supplementary material for: Acid-Sensing Ion Channels’ Immunoreactivity in Nerve Profiles and Glomus Cells of the Human Carotid Body
Source: Int J Mol Sci. 2023 Dec 5;24(24):17161. doi: 10.3390/ijms242417161 (PMC10743051; doi:10.3390/ijms242417161)
Supplement: Supplementary file 1 [file ijms-24-17161-s001.zip › ijms-2747402-supplementary.pdf]

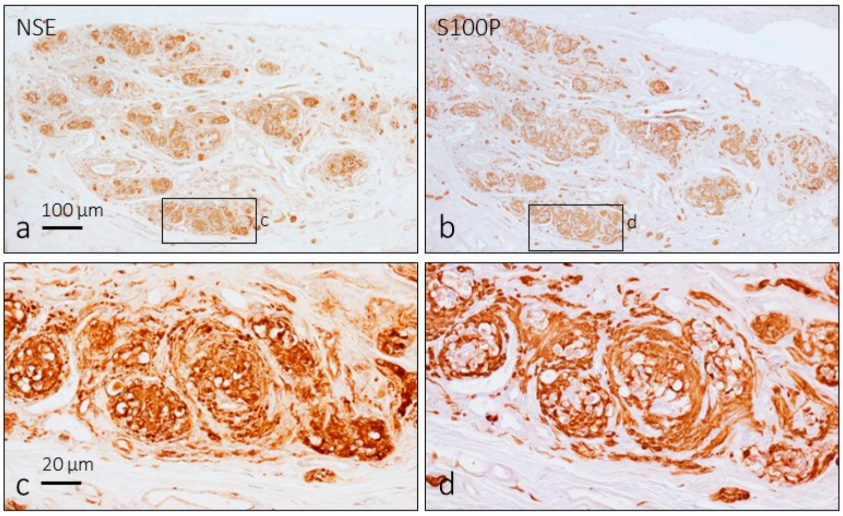

Figure S1. Immunohistochemical identification of type I glomus cells (a-c) and type II glomus cells (b-d) in the human carotid body. NSE: neuron-specific enolase; S100P: S100 protein.

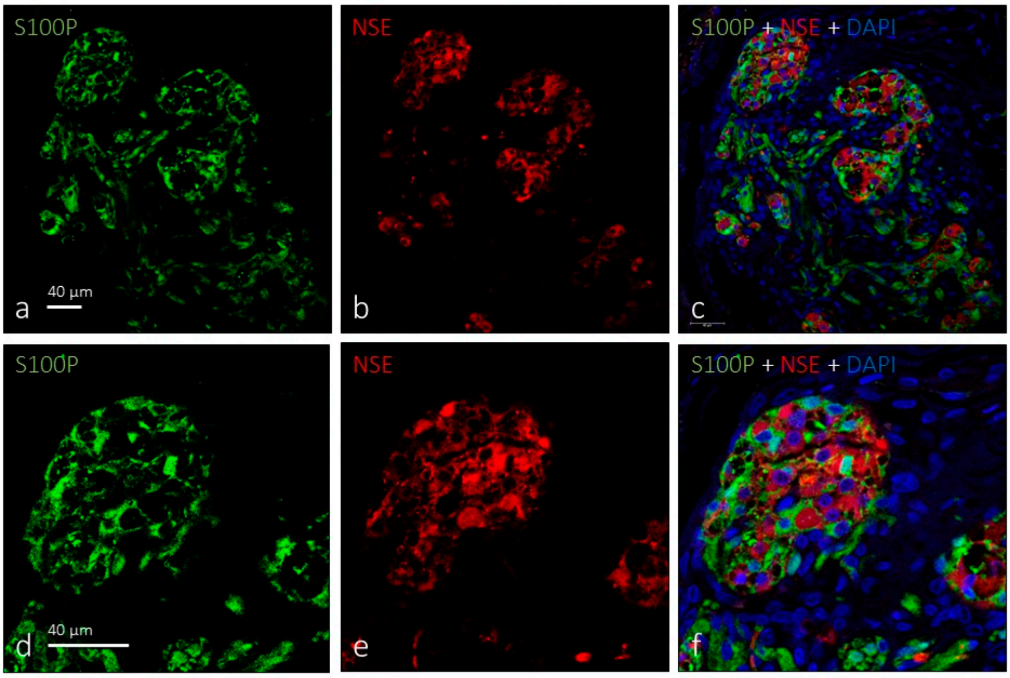

Figure S2. Identification of glomus cell types using immunofluorescence for NSE and S100P. The type I glomus cells displayed a strong immunofluorescence for neurons specific enolase (NSE, red fluorescence) while type II glomus cells were intensely immunolabelled by S100 protein (S100P, green fluorescence. Lens 60/1.25 oil; pinhole resolution 1.XY 156 nm and resolution Z 334 nm.

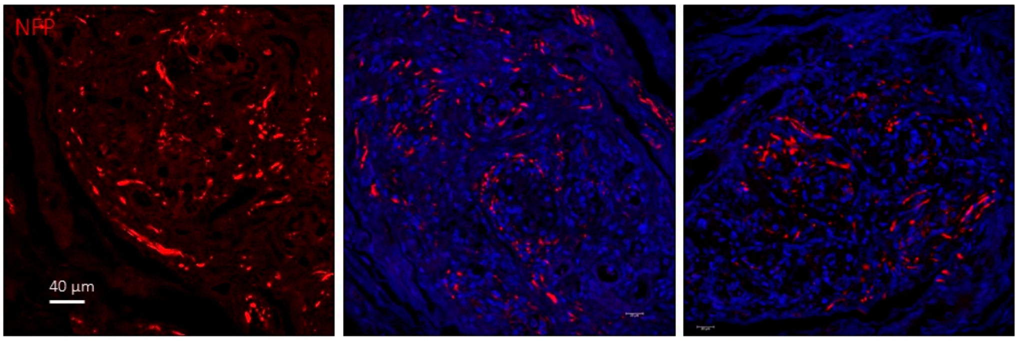

Figure S3. Distribution of nerve profiles in sections of the human carotid body immunolabelled for neurofilament proteins (NFP) Lens 60/1.25 oil; pinhole resolution 1.XY 156 nm and resolution Z 334 nm.

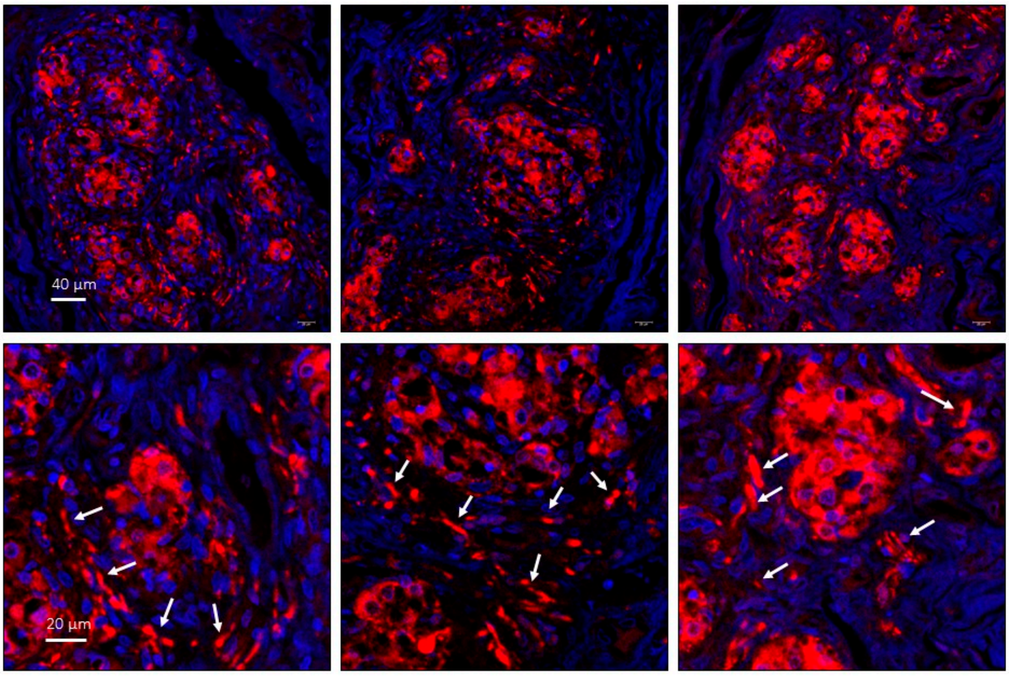

Figure S4. Identification of type I glomus cells and nerve profiles (white arrows) in the human carotid body immunolabelled for neuron specific enolase (NSE). Lens 60/1.25 oil; pinhole resolution 1.XY 156 nm and resolution Z 334 nm.
